# Altered Expression of Retinol Metabolism-Related Genes in an ANIT-Induced Cholestasis Rat Model

**DOI:** 10.3390/ijms19113337

**Published:** 2018-10-26

**Authors:** Kimitaka Takitani, Kanta Kishi, Hiroshi Miyazaki, Maki Koh, Hirofumi Tamaki, Akiko Inoue, Hiroshi Tamai

**Affiliations:** 1 Department of Pediatrics, Osaka Medical College, Osaka 569-8686, Japan; ped094@osaka-med.ac.jp (K.K.); silent-hero.m@osakah.johas.go.jp (H.M.); ped035@osaka-med.ac.jp (M.K.); tamaki@shinseikai.org (H.T.); ped031@osaka-med.ac.jp (A.I.); ped001@osaka-med.ac.jp (H.T.); 2 Department of Pediatrics, Osaka Rosai Hospital, Osaka 591-8025, Japan; 3 Department of Medicine, Shinseikai Daiichi Hospital, Aichi 468-0031, Japan

**Keywords:** cholestasis, retinol, bile acid, farnesoid X receptor

## Abstract

Cholestasis is defined as a reduction of bile secretion caused by a dysfunction of bile formation. Insufficient bile secretion into the intestine undermines the formation of micelles, which may result in the reduced absorption of lipids and fat-soluble vitamins. Here, we investigated the retinol homeostasis and the alterations of retinol metabolism-related genes, including β-carotene 15,15′ monooxygenase (BCMO), lecithin:retinol acyltransferase (LRAT), aldehyde dehydrogenase (ALDH), cytochrome P450 26A1 (CYP26A1), and retinoic acid receptors (RAR) β, in a α-naphthyl isothiocyanate (ANIT)-induced cholestasis rat model. Moreover, we examined the expression of the farnesoid X receptor (FXR) target genes. Our results showed that plasma retinol levels were decreased in ANIT rats compared to control rats. On the contrary, hepatic retinol levels were not different between the two groups. The expression of FXR target genes in the liver and intestine of cholestasis model rats was repressed. The BCMO expression was decreased in the liver and increased in the intestine of ANIT rats compared to control rats. Finally, the hepatic expression of LRAT, RARβ, and ALDH1A1 in cholestatic rats was decreased compared to the control rats, while the CYP26A1 expression of the liver was not altered. The increased expression of intestinal BCMO in cholestasis model rats might compensate for decreased circulatory retinol levels. The BCMO expression might be regulated in a tissue-specific manner to maintain the homeostasis of retinol.

## 1. Introduction

Retinol (vitamin A) is involved in many critical biological functions including vision, reproduction, immunity, cell differentiation/proliferation, and the development and growth of the embryo [1]. Retinol and protein deficiencies are currently the most severe and common nutritional diseases among children in developing countries [1]. Retinol deficiency is a cause of infant mortality in developing countries, leading to increased susceptibility to infections, which is a serious problem for the whole community. Retinol supplementation is used to prevent retinol deficiency and can reduce the mortality of children. 

Dietary retinol is obtained from animal-derived food, such as retinyl ester, or from plant sources, such as β-carotene [1]. Dietary retinyl esters are metabolized to retinol and are absorbed by the enterocytes together with β-carotene. Retinol and β-carotene are incorporated into chylomicrons and transferred to the liver. Residual β-carotene in enterocytes is converted to retinal by β-carotene oxygenases, including β-carotene 15,15′ monooxygenase (BCMO) and β-carotene 9,10′ monooxygenase [2]. BCMO is the predominant β-carotene cleaving enzyme and it has an essential role in retinol homeostasis. BCMO performs the central cleavage of β-carotene whereas β-carotene 9,10′ monooxygenase performs the eccentric cleavage of β-carotene. 

In the circulation, chylomicron remnants are recognized by lipoprotein lipases and taken up by hepatocytes [1]. In the hepatocytes, retinyl esters are hydrolyzed to retinol and they bind to the cellular retinol binding protein (CRBP)-I. Retinol bound to RBP, the predominant form of retinol in circulation, is then secreted in the blood. In stellate cells, a portion of retinol is stored following its esterification into retinyl ester by lecithin:retinol acyltransferase (LRAT). Retinol and retinal are reversibly interconverted by ester hydrolases and retinol dehydrogenases. Retinal can be oxidized to retinoic acid by members of the aldehyde dehydrogenase (ALDH) family. Eventually, retinoic acid is further processed by members of the cytochrome P450 family enzyme (CYP), including 26A1, 26B1 and 26C1, and the metabolites of retinoic acid biochemical transformations are conjugated with glucuronic acid for the excretion in urine and bile.

Retinoic acid receptors, including retinoic acid receptors (RARs) α, β, and γ; and retinoid X receptors (RXRs) α, β, and γ, are members of the nuclear receptor super family and act as ligand-dependent transcriptional factors. All-*trans* retinoic acid is the ligand of RARs and 9-*cis* retinoic acid is the ligand of RARs and RXRs. RAR and RXR form a heterodimer that binds to the response element located in the promoter of target genes. Thus, retinoic acid exerts its physiological actions through RARs and RXRs signaling pathway.

Cholestasis is defined as a reduction of bile secretion caused by either a dysfunction of bile formation at the hepatocellular level, or by an impaired secretion and flow of bile at the bile duct level [3,4]. When the secretion of substances including bilirubin, bile acids, and cholesterol into the bile is impaired, hepatocellular damage occurs. Cholestasis results in the accumulation of cytotoxic bile acids in the liver, leading to liver diseases, such as biliary fibrosis, cirrhosis and, ultimately, hepatic failure, requiring a transplant. Chronic cholestasis causes steatorrhea and malnutrition in adults and growth failure in children [5,6]. Bile acids are required for the micellar solubilization of dietary fat and fat-soluble vitamins (A, D, E, and K) in the intestine. Insufficient bile secretion into the intestine undermines the formation of micelles and may result in the reduced absorption of lipids and fat-soluble vitamins. Therefore, supplementation of fat-soluble vitamins is recommended for patients with chronic cholestatic liver diseases, particularly for neonates and infants because they have lower storage of fat-soluble vitamins at birth [7].

There are a number of reports investigating the metabolism of fat-soluble vitamins, including retinol, in cholestasis. Intrahepatic cholestasis is developed by several pathological conditions, including inherited disorders, drugs, pregnancy, primary biliary cirrhosis, and primary sclerosing cholangitis [8]. However, few studies focus on the alterations of retinol metabolism in intrahepatic cholestasis. There are several rodent models of human cholestatic liver diseases [9]. Bile duct ligation is well known as a model for extrahepatic cholestasis and develops secondary biliary fibrosis. Carbon tetrachloride is one of the most generally used toxins to induce liver injury and develops severe liver fibrosis. Alternatively, α-naphthylisothiocyanate (ANIT) induces the toxicity to hepatocytes and intra-hepatic bile epithelial cells, and produces a cholangiolitic hepatitis [10]. α-naphthylisothiocyanate (ANIT) is frequently used in rodents to generate intrahepatic cholestasis models [10]. Therefore, in this study, we investigate retinol homeostasis and the alteration of retinol metabolism-related proteins in an ANIT-induced cholestasis rat model.

## 2. Results

### 2.1. Biochemical Characterization of ANIT Rats

**Figure 1:**
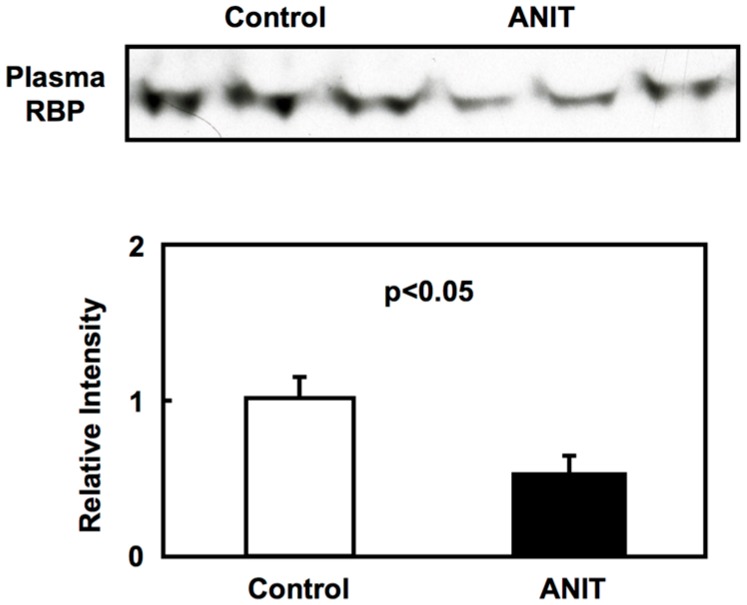
Plasma retinol binding protein (RBP) immunoblotting. Plasma samples from control rats and α-naphthylisothiocyanate (ANIT) rats (*n* = 3 for each group) were analyzed by immunoblotting to detect plasma RBP levels as described in Material and Methods. Each bar represents the mean value ± SD and significant differences are indicated in the graph.

ANIT rats showed higher total plasma bilirubin and alanine aminotransferase (ALT) levels compared with control rats, indicating cholestasis and hepatic dysfunction (Table 1). In ANIT rats, cholesterol levels in plasma were also higher than in controls, due to the reduced expression of the cholesterol-metabolizing enzyme [11,12]. Plasma retinol levels and RBP expression were significantly decreased in ANIT rats compared with control rats, while hepatic retinol levels were similar in both control and ANIT rats (Table 1 and Figure 1).

### 2.2. Gene Expression of Bile Acid-Metabolizing and Transporter Proteins 

**Figure 2:**
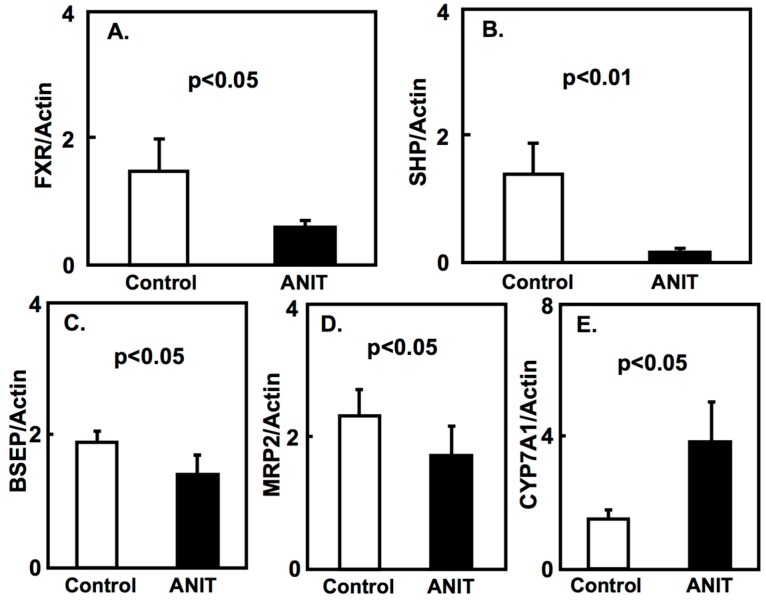
Hepatic expression of FXR, SHP, BSEP, MRP2, and CYP7A1 in both control and ANIT rats. Hepatic mRNA levels of FXR (**A**), SHP (**B**), BSEP (**C**), MRP2 (**D**), and CYP7A1 (**E**) in both groups (*n* = 5 for each group) were examined by quantitative real-time PCR. Each bar represents the mean value ± SD and significant differences are indicated in the graph.

**Figure 3:**
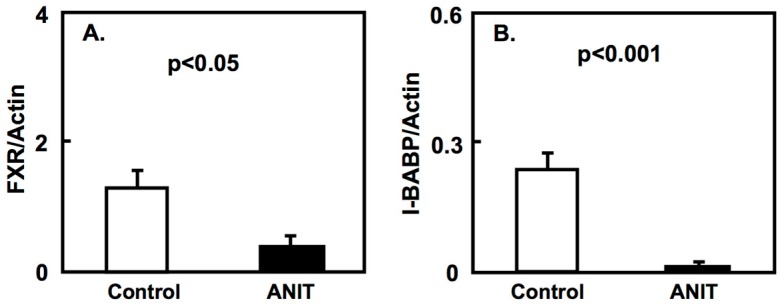
Intestinal expression of FXR and I-BABP in both control and ANIT rats. Intestinal mRNA levels of FXR (**A**) and I-BABP (**B**) in both groups (*n* = 5 for each group) were examined by quantitative real-time PCR. Each bar represents the mean value ± SD and significant differences are indicated in the graph.

We evaluated the expression of the farnesoid X receptor (FXR) and its target genes in a cholestasis rat model. FXR is the major regulator of bile acid homeostasis in the liver and intestine; it acts as a transcription factor and it regulates the expression of bile acid-metabolizing and transporter proteins [13]. Our results showed that the expression of FXR, small heterodimer partner (SHP), bile salt export pump (BSEP), and multidrug resistance protein-2 (MRP2) in the liver of ANIT rats was lower compared with control rats. On the contrary, the hepatic expression of CYP7A was higher in ANIT rats than in control rats (Figure 2). Furthermore, the intestinal expression of FXR and intestinal bile acid-binding protein (I-BABP) in ANIT rats was lower than that in control rats (Figure 3).

### 2.3. Gene Expression of BCMO, LRAT, and RARβ in Peripheral Tissues

**Figure 4:**
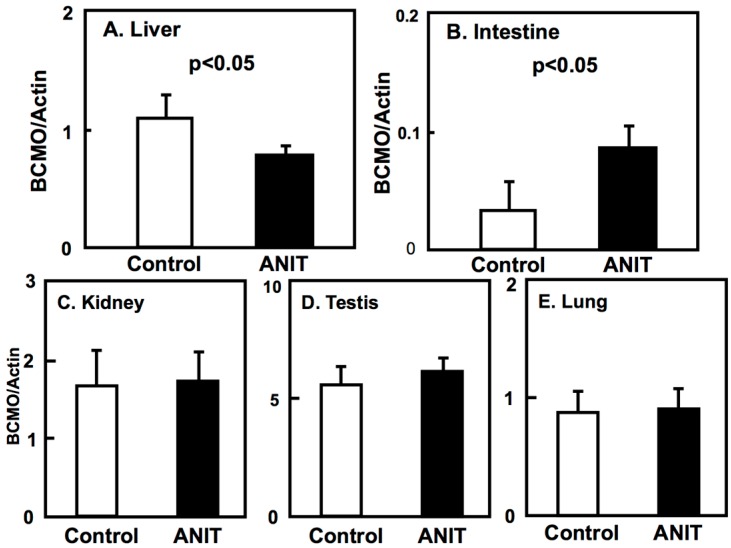
Tissue-specific expression of BCMO in control and ANIT rats. The BCMO mRNA levels of peripheral tissues including liver (**A**), intestine (**B**), kidney (**C**), testis (**D**), and lung (**E**) were examined in treated and controls groups (*n* = 5 for each group) by quantitative real-time PCR. Each bar represents the mean value ± SD and significant differences are indicated in the graph.

**Figure 5:**
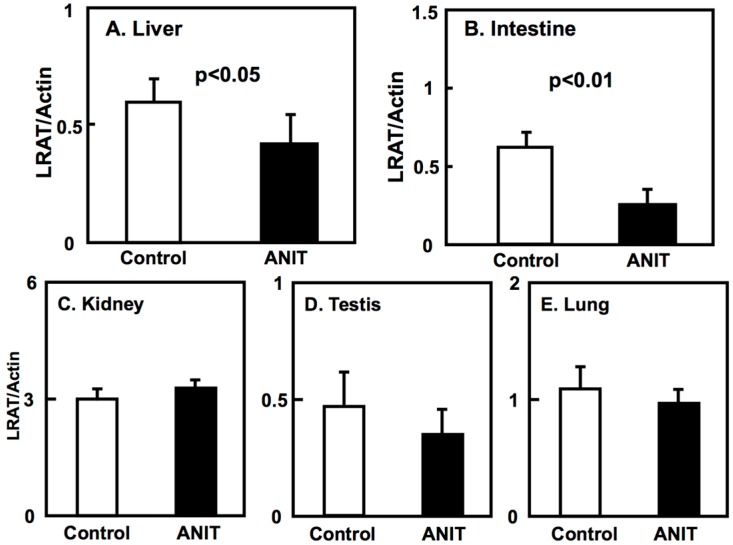
Tissue-specific expression of LRAT in control and ANIT rats. The LRAT mRNA levels of peripheral tissues including liver (**A**), intestine (**B**), kidney (**C**), testis (**D**), and lung (**E**) were examined in treated and control groups (*n* = 5 for each group) by quantitative real-time PCR. Each bar represents the mean value ± SD and significant differences are indicated in the graph.

**Figure 6:**
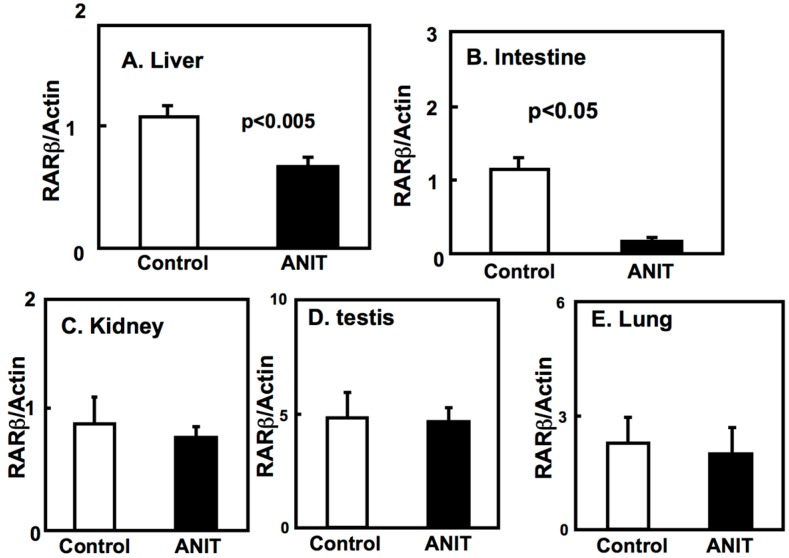
Tissue-specific expression of RARβ in control and ANIT rats. RARβ mRNA levels of peripheral tissues including liver (**A**), intestine (**B**), kidney (**C**), testis (**D**), and lung (**E**) were examined by quantitative real-time PCR in treated and control groups (*n* = 5 for each group). Each bar represents the mean value ± SD and significant differences are indicated in the graph.

Next, we examined the expression of BCMO, LRAT, and RARβ in peripheral tissues including liver, intestine, kidneys, testes, and lungs (Figure 4, Figure 5 and Figure 6). The BCMO expression in the liver of ANIT rats was significantly lower compared to controls, while its expression in the intestine of ANIT rats was markedly increased compared to controls (Figure 4). The BCMO expression in the other tissues considered did not vary among controls and ANIT rats. The LRAT and RARβ expression in the liver and intestine of ANIT rats was lower compared to controls and it did not differ among the two groups in the other tissues considered (Figure 5 and Figure 6). 

### 2.4. Gene Expression of CRBPI, ALDH1As, and CYP26A1 in the Liver, and ALDH1As Expression in Peripheral Tissues

**Figure 7:**
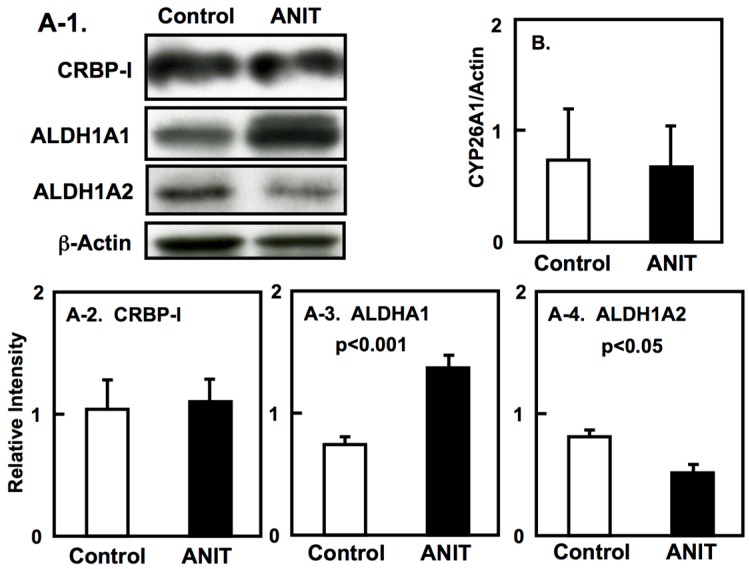
Hepatic expression of ALDH1A1, ALDH1A2, CRBP-I and CYP26A1 in control and ANIT rats. (**A**) Hepatic protein levels of CRBP-I (A-1, 2), ALDH1A1 (A-1, 3), ALDH1A2 (A-1, 4), and in control and ANIT rats (*n* = 3 for each group) were analyzed by immunoblotting using suitable antibodies. Hepatic β-actin was used as the loading control. (**B**) The hepatic mRNA levels of CYP26A1 in both groups (*n* = 5 for each group) were examined by quantitative real-time PCR. Each bar represents the mean value ± SD and significant differences are indicated in the graph.

**Figure 8:**
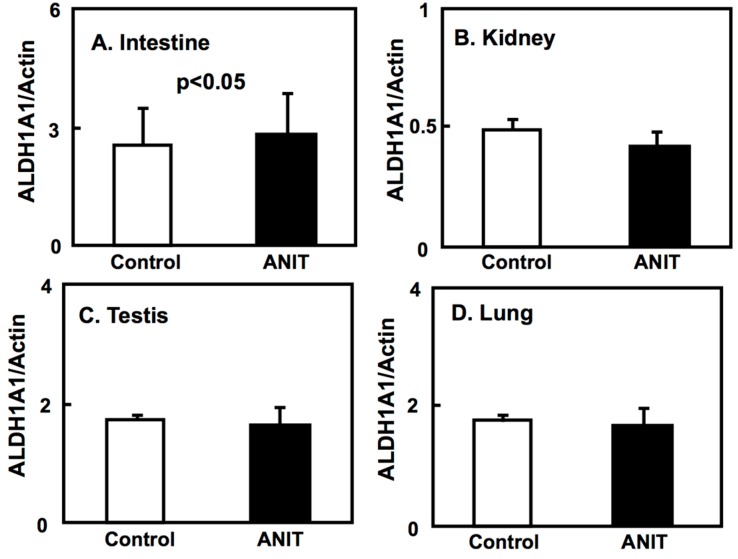
Tissue-specific expression of ALDH1A1 in control and ANIT rats. The mRNA levels of the ALDH1A1 gene of several tissues including intestine (**A**), kidney (**B**), testis (**C**), and lung (**D**) in both groups (*n* = 5 for each group) were examined by quantitative real-time PCR. Each bar represents the mean value ± SD and significant differences are indicated in the graph.

**Figure 9:**
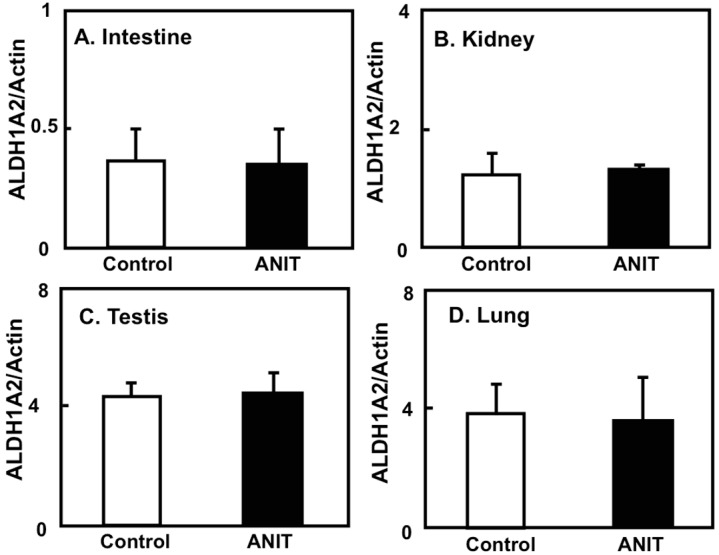
ALDH1A2 gene expression of several tissues in both control and ANIT rats. The ALDH1A2 mRNA levels of tissues including intestine (**A**), kidney (**B**), testis (**C**), and lung (**D**) were examined by quantitative real-time PCR in treated and control groups (*n* = 5 for each group). Each bar represents the mean value ± SD and significant differences are indicated in the graph.

We elucidated the tissue-specific expression of retinol-related proteins, including metabolic enzymes and binding proteins. The expression of CRBP-I and CYP26A1 in the liver of ANIT rats was unaltered compared to control rats (Figure 7A-1,A-2,B). The hepatic expression of ALDH1A1 in ANIT rats was increased compared to control rats while the hepatic expression of ALDH1A2 was decreased in ANIT rats compared to control rats (Figure 7A-1,A-3,A-4). However, the expression of both ALDH1A1 and ALDH1A2 in the other tissues considered was not altered in control and ANIT rats (Figure 8 and Figure 9). 

## 3. Discussion

In physiological conditions, FXR is expressed in the liver, intestinal villi, renal tubes, and adrenal cortex. FXR knock out mice showed impaired bile acid or lipid metabolism [14]. In the current study, the expression of FXR and other target genes including SHP, BSEP, and MRP2, showed a decrease in the liver of ANIT rats compared to control rats. On the contrary, the expression of hepatic CYP7A1 showed an increase compared to control rats. Moreover, the expression of intestinal FXR and its target, I-BASP, was decreased in ANIT rats. These findings suggest that the FXR signaling pathway is impaired in the cholestasis rat model, in agreement with previous studies [15,16,17]. The expression of CYP7A1 in the liver is repressed through FXR signaling [11,12]; therefore, its reduced expression in the liver of ANIT rats may be due to the lower expression of FXR. In the previous experiments using ANIT-induced hepatic injury rodents, the expression of FXR-related genes, including enzymes and transporters, was altered in a time-dependent manner [18,19]. However, in the current study, the duration of treatment with ANIT is four days and the experiment was performed at a single point in time. Therefore, this experiment design may be insufficient to evaluate significant FXR-mediated effects. 

Several studies have focused on retinol deficiency and human cholestatic liver diseases [20]. In patients with primary biliary cholangitis or primary sclerosing cholangitis, conflicting findings have been reported with respect to serum retinol levels; the retinol levels of cholestatic patients were decreased or not altered compared with control subjects [21]. However, it was reported that serum retinol levels are associated with biochemical parameters including albumin, total cholesterol, triglycerides, total bilirubin, and aspartate transaminase, and the hepatic histological findings of patients with primary biliary cholangitis. Therefore, variations in retinol levels may reflect different stages of cholestatic liver disease. In an animal study, performed in a bile duct-ligated cholestasis rat model, the combination treatment of retinoic acid with ursodeoxycholic acid improved liver fibrosis [22]. Alternatively, in a clinical study that examined the combination therapy of all-*trans* retinoic acid with ursodeoxycholic acid in patients with primary sclerosing cholangitis, the levels of ALT and of a bile acid synthetic precursor (7α-hydroxy-4-cholesten-3-one) were decreased. However, alkaline phosphatase (ALP) levels did not decrease significantly [23]. This clinical study did not attain the primary endpoint (reduced ALP levels). Further investigations on all-*trans* retinoic acid therapy for cholestatic liver diseases are required in the future. 

In the present study, the retinol concentration in the liver was the same in control and ANIT rats. However, the plasma levels of retinol and RBP expression were markedly decreased in ANIT rats. This result may be observed due to the duration of the experimental study. Rats were sacrificed four days after ANIT administration; in the considered time-frame, hepatic retinol levels may have not yet been affected by ANIT administration, despite reduced hepatic LRAT expression. However, the impaired expression of hepatic LRAT in ANIT rats might lead to retinol storage in the liver as well as to the development of cholestasis. The decreased expression of LRAT in the liver in the cholestasis rat model may enhance the impaired storage of retinol. 

BCMO is the primary enzyme metabolizing β-carotene to retinal, and it is largely responsible for maintaining retinol homeostasis [24]. The regulation of BCMO expression is controlled by the homeobox transcriptional factor intestine specific homeobox (ISX), diet, and supplementation with retinol or β-carotene [24,25,26]. We previously reported that BCMO expression is altered in several peripheral tissues in pathological conditions including nephrosis, obesity with dyslipidemia, type 1 diabetes, type 2 diabetes, and non-alcoholic fatty liver disease (NAFLD); moreover, it can also be altered by biological substances such as dehydroepiandrosterone [27,28,29,30,31,32]. From these findings, we considered that the retinol levels in blood and the BCMO expression in the liver and intestine may affect one another; namely, it is possible that the BCMO expression can be altered in the kidney, testis, and lung to compensate for reduced circulatory retinol levels due to a pathological condition. In the current study, the hepatic expression of BCMO was increased in ANIT-induced cholestasis rats and its intestinal expression was higher compared to controls rats. In other peripheral tissues, including the kidney, testis, and lung, BCMO expression was not altered. Reduced BCMO expression in the liver may be caused by cholestatic liver injury, and higher expression in the intestine might compensate for the decreased circulatory retinol levels. BCMO gene expression might be regulated in a tissue-specific manner to maintain retinol homeostasis. Further investigations should be conducted to ascertain the regulatory mechanisms of BCMO expression in different tissues.

The CYP26 family consists of three subtypes: CYP26A1, CYP26B1, and CYP26C1. These enzymes metabolize retinoic acid into 5-hydroxy retinoic acid, 4-oxo retinoic acid, and 18-hydroxy retinoic acid, and their expression is enhanced by retinoic acid [33,34]. The promoter region of CYP26A1 contains a retinoic acid response element (RARE), and its expression is regulated by RAR/RXR signaling [35]. Moreover, CRBP-I and RARβ contain a RARE in their promoter region, as well as the target genes of RAR/RXR [36,37]. LRAT-mediated retinol conversion into retinyl ester has a critical role in the storage of dietary retinol [38]. Even though the promoter of the LRAT gene in rats and humans lacks the RARE in its proximal 5′ flanking region, LRAT expression is still enhanced by retinol or retinoic acid [38,39]. Therefore, LRAT is considered a retinoic acid-responsive gene. In the present study, the hepatic expression of CRBP-I and CYP26A1 was found to vary between the ANIT and control rats; however, LRAT and RARβ expression in the liver and intestine was found to decrease in ANIT rats compared to control rats. Therefore, transcriptional signaling via RAR/RXR in the liver and intestine might be impaired under a cholestatic condition. 

Three types of cytosolic ALDH1A (ALDH1A1, ALDH1A2, and ALDH1A3) play a role in oxidizing retinaldehyde to retinoic acid, which is a ligand for the nuclear receptors RAR and RXR [40]. ALDH1A1 is highly expressed in the dorsal retina of embryos and in several epithelial tissues in adult rodents. ALDH1A2 is conserved during development [41]. ALDH1A3 is expressed in the eye structures, as well as in the olfactory and urinary tracts. It is reported that the expression pattern of ALDH1A1 in different tissues is regulated by several factors, including sex hormones and lipids [42]. The current study showed that while the hepatic ALDH1A1 expression increases, that of hepatic ALDH1A2 decreases in ANIT rats compared to control rats. The expression of both ALDH1A1 and ALDH1A2 in the other peripheral tissues was not different between the treated and control groups. This expression pattern is consistent with a previous report, in which we investigated the BCMO expression of type-1 diabetes model rats [31]. In a type 1 diabetes rat model, rats presented lower plasma retinol levels and higher hepatic retinol levels compared to the control rats, as well as lower and higher expression of ALDH1A1 and ALDH1A2, respectively. The mechanism underlying this expression pattern remains to be elucidated; however, increased hepatic ALDH1A1 expression in ANIT rats may compensate for the suppression of RAR signaling, while hepatic ALDH1A2 expressing rats might be more susceptible to cholestatic liver injury than those expressing ALDH1A1. Further studies will be necessary to clarify this issue.

In the present study, we have focused on retinol metabolism under the pathological condition of cholestasis and revealed the dysregulation of retinol metabolism including metabolic enzymes. As mentioned above, hepatic expression of several retinol metabolism-related genes was impaired in cholestatic model rats. Several reports demonstrate that retinoic acid, as well as FXR agonists, have the effect on cholestasis [43,44]. Retinoic acid treatment ameliorates serum levels of hepatic enzymes and reduces bile duct proliferation and inflammation in cholestatic model rodents [45,46]. RARα and RXRα expression was diminished in cholestatic liver injury, therefore retinoic acid signaling is impaired [47,48]. Moreover, Weiss reported that retinol deficiency may exacerbate cholestasis due to the enhanced proliferation of bile duct epithelial cells [49]. These previous findings may support the dysregulated retinol metabolism in cholestasis.

In the current study, several limitations must be noted. First, the experimental setting is largely static and does not reflect the dynamics of gene and protein expression in this ANIT-induced cholestasis rat model. Second, in vitro investigations and especially in situ investigations are missing. As mentioned above, retinoic acid, a metabolite of retinol, is considered to have a potentially beneficial effect for human cholestatic liver diseases [20,23]; therefore, the current study might contribute to the mechanism of retinoic acid therapy for cholestasis. To clarify this issue, it is better to evaluate the effect of retinoic acid on the expression of retinol metabolism-related genes for comparison.

In conclusion, in this study, we investigated the tissue-specific presence of retinol and the expression of retinol metabolism-related proteins in an ANIT-induced cholestasis rat model. The expression of FXR target genes in both the liver and intestine was repressed in cholestasis model rats. The expression of BCMO, LRAT, and RARβ was decreased in the liver of ANIT rats, which may be due to the cholestatic liver injury. Moreover, the increased expression of intestinal BCMO in cholestatic model rats might compensate for decreased circulatory retinol levels. The BCMO expression might be regulated in a tissue-specific manner to maintain retinol homeostasis. Further investigations will be required to clarify the mechanism behind these expression patterns. 

## 4. Materials and Methods 

### 4.1. Animal Experiments

Four-week-old male Wistar rats were obtained from Japan SLC Inc. (Sizuoka, Japan) and were subjected to a standard diet. The food was supplemented with retinol (10,000 IU/kg) and β-carotene (0.2 mg/kg) and it was purchased from Funabashi Farm (Chiba, Japan) [26]. The care and handling of animals was performed according to Osaka Medical College guidelines for the ethical treatment of laboratory animals (approval No. 25031 on 21 May 2013). Rats were divided into two groups of five animals each, producing a control group and an ANIT-administered group. On day 10, rats in the ANIT group were orally administered a single dose of 100 mg/kg body weight of ANIT (Wako Pure Chemical Industries, Ltd., Osaka, Japan) in olive oil formulation whereas the control rats were administered the same dose of formulation without ANIT. After an overnight fast, the rats were sacrificed on day 14 by exsanguination under isoflurane anesthesia. Blood was collected into heparinized tubes and plasma was stored at −80 °C. Tissues including the liver, the intestine (jejunum), the lungs, the kidneys and the testes were removed, then immediately frozen in liquid nitrogen and stored at −80 °C.

### 4.2. Biochemical Data Analysis

Plasma levels of cholesterol, ALT, and total bilirubin were determined by the enzymatic colorimetric method. Retinol levels in the plasma and liver homogenates were assayed using high-performance liquid chromatography, as described previously [50]. The liver tissue was homogenized and subsequently saponified with a one-twentieth volume of 60% potassium hydroxide in distilled water. Then, the saponified liver samples were extracted with hexane and the protein content was measured according to the Bradford method [51].

### 4.3. Immunoblotting

Homogenized protein samples from the liver were prepared for immunoblotting as described previously [28]. The extracted proteins were electrophoresed and transferred to a PVDF membrane. Non-specific binding sites on the membrane were blocked by 5% non-fat milk in Tris-buffered saline containing Tween-20 (TBS-T). Antibodies specific for cellular retinol binding protein (CRBP)-I (Santa Cruz Biotechnology Inc., Dallas, TX, USA), ALDH1A2 (Santa Cruz Biotechnology Inc.), ALDH1A1 (ProSci Inc., Poway, CA, USA), and β-actin (Medical & Biological Laboratories Co. Ltd., Nagoya, Japan) were used as the primary antibodies. The appropriate horseradish peroxidase-conjugated secondary antibodies (Bio-Rad Laboratories, Hercules, CA, USA) were used for the detection of each protein, and the signals of the target proteins were detected with the ECL Western Blotting Detection System (GE Healthcare UK Ltd., Little Chalfont, UK). The intensity of the protein bands on immunoblotting images was measured using the ImageJ 1.46r software (National Institute of Mental Health, Bethesda, MD, USA). The ratio of the intensity of each protein band to that of a -actin standard was determined, and the mean and standard deviation of the ratios were calculated. An anti-human RBP antibody (Santa Cruz Biotechnology Inc.) was used to evaluate the plasma RBP levels, as described previously [24].

### 4.4. Reverse Transcription and Quantitative Real-Time PCR

Total RNA was isolated from frozen tissues using ISOGEN (Fujifilm Wako Pure Chemical Corp., Ltd., Osaka, Japan). Subsequently, reverse transcription was carried out using Omniscript (Qiagen, Hilden, Germany) according to the manufacturer’s instructions. To determine the level of expression for each gene, quantitative real-time PCR was performed. Subsequently, 2 µL of each RT reaction mixture was amplified by LightCycler PCR (F. Hoffmann-La Roche Ltd. Diagnostics Division, Basel, Switzerland) using a LightCycler-FastStart DNA Master Hybridization Probe Kit, or FastStart DNA Master SYBR Green I Kit, according to the manufacturer’s instructions (F. Hoffmann-La Roche Ltd. Diagnostics Division). The sequences of oligonucleotide primer sets and their accession numbers are listed in Table 2 for the following proteins: aldehyde dehydrogenase 1A (ALDH1A), β-carotene 15,15′ monooxygenase (BCMO), bile salt export pump (BSEP or ABCB11), -actin, cholesterol 7α-hydroxylase (CYP7A1), cytochrome P450 26A1 (CYP26A1), farnesoid X receptor (FXR), intestinal bile acid-binding protein (I-BABP), lecithin:retinol acyltransferase (LRAT), multidrug resistance protein-2 (MRP2 or ABCC2), retinoic acid receptor β (RARβ), and small heterodimer partner (SHP). Rat *BCMO* and *β-actin* were analyzed using a Master Hybridization Probe Kit while the other genes were evaluated using a Master SYBR Green I Kit. Rat *β-actin* was used as a reference for normalizing the gene copy numbers of each sample.

### 4.5. Statistical Analysis

All results were expressed as mean ± standard deviation (SD). Differences between groups were tested for statistical significance using Welch’s *t*-test. Differences between groups were considered significant at a *p*-value of less than 0.05. 

## Figures and Tables

**Table 1:** Biochemical profiles and retinol status between control and ANIT rats.

	Control	ANIT
	(*n* = 5)	(*n* = 5)
Total cholesterol (mg/dL)	66.8 ± 3.2	218.8 ± 18.1 ***
Total Bilirubin (mg/dL)	0.04 ± 0.01	0.53 ± 0.3 *
ALT (U/L)	35.8 ± 3.3	376.2 ± 180.4 *
Retinol (plasma) (µg/dL)	50.8 ± 4.6	35.6 ± 3.4 ***
Retinol (liver) (µg/mg protein)	0.17 ± 0.02	0.15 ± 0.02

Values are expressed mean ± SD and asterisks indicate significant differences (* *p* < 0.05, *** *p* < 0.001) between control and ANIT rats.

**Table 2:** Sequences of oligonucleotide primers for real-time PCR.

Gene (Accession Number)	Forward	Reverse	Length (bp)
ALDH1A1 (BC061526)	5′-ATGGTCTAGCAGCAGGAGT-3′	5′-CCAGACATCTTGAATCCACCGAA-3′	142
ALDH1A2 (NM_053896)	5′-TTGCCTCACAACAAGTGAGC-3′	5-ACAAAATGGGGTTCATTGGA-3'	125
BCMO (NM_053648)	5′-CAAGTCCTCCTTAAAGTGGT-3′	5′-AATAAACCATGCAGGTCCA-3′	225
	5′-CATCATCTCTACAGATCCCCAAAAGC-3′-FITC		
	LC-5′-GCCCTTTTTACTTATTCTGGATGCG-3′		
BSEP (NM_031760)	5′-GCCATTGTGCGAGATCCTAAA-3′	5′-TGCAGGTCCGACCCTCTCT-3′	118
β-actin (V01217 J00691)	5′-CCTGTATGC CTC TGG TCG TA-3′	5′-CCATCTCTTGCTCGAAGTCT-3′	260
	5′-CGGGACCTGACAGACTACCTCATG-3′-FITC		
	LC-5′-AGATCCTGACCGAGCGTGGCTAC-3′		
CYP7A1 (NM_012942)	5′-GCTTTACAGAGTGCTGGCCAA-3′	5′-CTGTCTAGTACCGGCAGGTCATT-3′	92
CYP26A1 (DQ266888)	5′-GTGCCAGTGATTGCTGAAGA-3′	5'-AGAGAAGAGATTGCGGGTCA-3′	213
FXR (U18374)	5′-GTGACAAAGAAGCCGCGAAT-3′	5′-GCAGGTGAGCGCGTTGTAAT-3′	114
I-BABP (L22788)	5′-CACTATGGCCTTCACCGGCAAATA-3′	5-ACCCTCCATCTTCACGGTTGCCTT-3′	268
LRAT (AF255060)	5′-GGAACAACTGCGAACACT-3′	5′-ACACTAATCCCAAGACAGC-3′	141
MRP2 (NM_012833)	5′-CTGGTGTGGATTCCCTTGG-3′	5′-CAAAACCAGGAGCCATGTGC-3′	252
RARβ (NM_031529)	5′-ATACCCCAGAGCAAGACACC-3′	5′-AGCAGATGGCACTGAGAAGA-3′	170
SHP (D86839)	5′-CTCGGTTTGCATACAGTGTTTGAC-3′	5′-GCATATTGGCCTGGAGGTTTT-3′	75

## Abbreviations

ALT	alanine aminotransferase
ALDH	aldehyde dehydrogenase
ANIT	α-naphthyl isothiocyanate
BCMO	β-carotene 15,15′ monooxygenase
BSEP	bile salt export pump
CRBP	cellular retinol binding protein
FXR	farnesoid X receptor
I-BABP	intestinal bile-acid binding protein
LRAT	lecithin-retinol acyltransferase
MRP2	multidrug resistance protein-2
NAFLD	non-alcoholic fatty liver disease
RARE	retinoic acid response element
RAR	retinoic acid receptor
RXR	retinoid X receptor
RBP	retinol binding protein
CYP7A1	7α-hydroxylase
SHP	small heterodimer partner
